# Drug–drug interaction extraction via hierarchical RNNs on sequence and shortest dependency paths

**DOI:** 10.1093/bioinformatics/btx659

**Published:** 2017-10-25

**Authors:** Yijia Zhang, Wei Zheng, Hongfei Lin, Jian Wang, Zhihao Yang, Michel Dumontier

**Affiliations:** 1 College of Computer Science and Technology, Dalian University of Technology, Dalian, China; 2 Stanford Center for Biomedical Informatics Research, School of Medicine, Stanford University, Stanford, CA, USA; 3 College of Software, Dalian JiaoTong University, Dalian, China; 4 Institute of Data Science, Maastricht University, Maastricht, ER, The Netherlands

## Abstract

**Motivation:**

Adverse events resulting from drug-drug interactions (DDI) pose a serious health issue. The ability to automatically extract DDIs described in the biomedical literature could further efforts for ongoing pharmacovigilance. Most of neural networks-based methods typically focus on sentence sequence to identify these DDIs, however the shortest dependency path (SDP) between the two entities contains valuable syntactic and semantic information. Effectively exploiting such information may improve DDI extraction.

**Results:**

In this article, we present a hierarchical recurrent neural networks (RNNs)-based method to integrate the SDP and sentence sequence for DDI extraction task. Firstly, the sentence sequence is divided into three subsequences. Then, the bottom RNNs model is employed to learn the feature representation of the subsequences and SDP, and the top RNNs model is employed to learn the feature representation of both sentence sequence and SDP. Furthermore, we introduce the embedding attention mechanism to identify and enhance keywords for the DDI extraction task. We evaluate our approach using the DDI extraction 2013 corpus. Our method is competitive or superior in performance as compared with other state-of-the-art methods. Experimental results show that the sentence sequence and SDP are complementary to each other. Integrating the sentence sequence with SDP can effectively improve the DDI extraction performance.

**Availability and implementation:**

The experimental data is available at https://github.com/zhangyijia1979/hierarchical-RNNs-model-for-DDI-extraction.

**Supplementary information:**

Supplementary data are available at *Bioinformatics* online.

## 1 Introduction

A drug-drug interaction (DDI) occurs when one drug influences on the level or activity of another co-administered drug (Miranda *et al.*, 2011). DDIs can delay or decrease absorption of drugs, and may cause severe adverse drug reactions (ADRs). When a patient administers multiple drugs together, there is an inevitable risk of DDIs. Some serious unexpected ADRs will be life-threatening or even cause death. Although some drug knowledge database, such as DrugBank (Knox *et al.*, 2011), PharmGKB (Thorn *et al.*, 2013), Drug Interaction database (Hachad *et al.*, 2010) and SFINX (Böttiger *et al.*, 2009), have been created to instruct physicians to avoid DDIs and ADRs, the update periods of these databases are generally 1–3 years. DDIs are frequently reported in the biomedical literature and may prove to be a valuable source of DDI information. Hence, the automatic extraction of DDIs information from the biomedical literature has merit and may contribute significantly to patient safety and pharmacovigilance (Percha and Altman, 2013).

In recent years, several efforts have been made in DDI extraction from the biomedical literature. Various existing methods can be mainly divided into two categories: statistical machine learning-based methods and neural networks-based methods.

One major approach is statistical or machine learning-based methods. Various of lexical and syntactic features are extracted and supply to the classifier. (Björne *et al.*, 2013) exploited shortest path features and domain knowledge features to extract DDI. (Kim *et al.*, 2015) proposed a rich feature-based method to extraction DDI, which including word features, dependency graph features, parse tree features, etc. Similarly, (Raihani and Laachfoubi, 2016) integrated lexical features, phrase features, verb features, syntactic features and auxiliary features to extract DDI from biomedical literature. In the feature-based methods, the major challenge is how to choose the suitable lexical and syntactic feature for the DDI extraction task. Up to now, feature extraction is still a time-consuming and skill-dependent task.

Since the syntactic parse tree and dependency graph carry important syntactic information for relation extraction task, some kernel methods have been proposed and successfully used for DDI extraction. (Zhang *et al.*, 2012) proposed hash subgraph pairwise kernel method to extraction DDI, which can effectively capture syntactic information of the dependency graph. (Chowdhury and Lavelli, 2013) proposed a hybrid kernel method for DDI extraction including feature-based kernel, shallow linguistic kernel and path-enclosed tree kernel. The hybrid kernel method achieved an *F*-score of 0.651 and the top rank in the DDI extraction 2013 challenge. (Thomas *et al.*, 2013) also employed multiple kernel methods and used majority voting-based model to detect DDI, which ranked as the second in the DDI extraction 2013 challenge. In general, these kernel-based methods can make better use of syntactic information in the dependency graph and syntactic parse tree than feature-based methods. However, the suitable kernel functions require carefully crafting, which have been proved difficult because of the powerful expressiveness of graph or tree structures (Gärtner *et al.*, 2003). Therefore, the performance of statistical machine learning-based methods is highly dependent on the chosen feature set or the designed kernel function.

Deep neural networks have emerged as promising approaches for automatic feature learning and have become a dominant method for DDI extraction task. Convolutional neural networks (CNNs) can effectively learn the local features through discrete convolution with different size filters. Some CNNs-based methods have been applied to extract DDI successfully. (Liu *et al.*, 2016) used CNNs model to extract DDI with the word and position embedding, and achieved an *F*-score of 0.698 on the DDI extraction 2013 corpora. (Quan *et al.*, 2016) proposed a multichannel CNNs model for DDI extraction task, which fused five version word embedding. (Zhao *et al.*, 2016) attempted to train word embedding based on syntax information and employed CNNs model to detect DDI from biomedical literature. Recurrent neural networks (RNNs) are another common neural networks. Compared to CNNs, RNNs are temporal sequence models and good at capturing the sentence sequence feature, which is consider to be more suitable for natural language processing (NLP) tasks. In the most recent, (Sahu and Anand, 2017) employed RNNs model with attention pooling method to extract DDI, and achieved better performance than CNNs-based methods.

DDI extraction 2013 challenge provided an opportunity to evaluate the performance of the various DDI extraction methods on the same benchmark corpora. So far, the best performance of DDI extraction is still <0.75 in *F*-score. The key challenge remains in how to accurately detect and classify the DDI in the complicated biomedical sentences. For example, the longest sentence in DDI extraction 2013 corpora contains >150 words. The length of such sentences are very hard to deal with for deep neural networks. A recent study (Mingguang Xiao, 2016) has shown dividing a sentence into multiple parts according to the entities present can boost the performance of relation extraction effectively. On the other hand, shortest dependency paths (SDP) are informative to determine the DDI in the sentences (Xu *et al.*, 2015). Most of neural networks-based methods only use the sentence sequence, but do not take full advantage of the valuable information of SDP. Incorporating the SDP information will be beneficial for deep neural networks to extract DDI, particularly for the complicated sentences.

In this article, we explore the effectiveness of SDP for extracting drug-drug interactions. Inspired by the work of (Mingguang Xiao, 2016), the sequence sentence is divided into three context subsequences according to the two candidate drug entities. Attention mechanism has been proven to be helpful in boosting the performance of NLP tasks (Wang *et al.*, 2016). We exploit embedding attention mechanism to identify and enhance keywords for the DDI extraction task. Then we integrate the context subsequences and SDP of the sentence, and employ hierarchical bidirectional RNNs model to automatically learning the latent feature from the both sequence and SDP structure. The bottom RNNs learn the local context representation of subsequence context and the syntax representation of SDP, respectively. The top RNNs learn the sentence representation for DDI extraction from the subsequence context and syntax representations. Softmax function is applied in the output layer to implement DDI detection and classification. Finally, our proposed model is evaluated on the DDI extraction 2013 corpus. Experimental results show that the syntactic and semantic information of SDP are valuable for DDI extraction. Our method can effectively integrate the sequence and SDP for DDI extraction, and achieve the state-of-the-art performance on DDI extraction 2013 corpus.

## 2 Materials and methods

DDI extraction task is generally tackled as the task of identifying the semantic relation holding between the two drugs among a set of candidate relations. According to the DDI extraction 2013 challenge, these candidate relations include *Negative*, *Advice*, *Effect*, *Mechanism*, *Int*. In this section, we first introduce the value of the SDP for DDI extraction task. Then, our DDI extraction model is described in detail.

### 2.1 Shortest dependency path

**Fig. 1.**
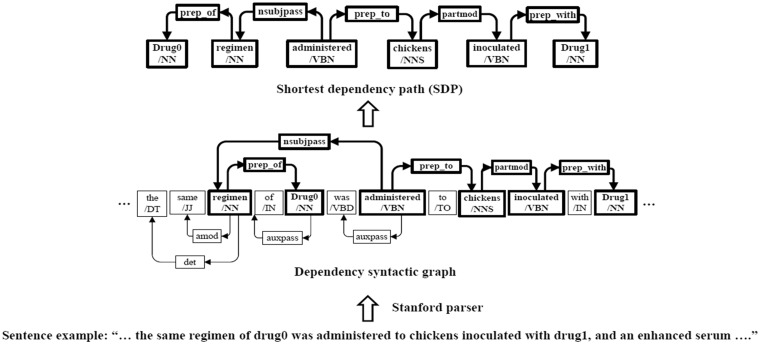
An illustration of SDP. The sentence example is from DDI extraction 2013 corpus. ‘Drug0’ and ‘Drug1’ denote two targeted drug entities, respectively. The Stanford parser is used to syntactic parse the sentence and generate the dependency syntactic graph. The nodes and edges on the shortest path between ‘Drug0’ and ‘Drug1’ are shown in bold. SDP between ‘Drug0’ and ‘Drug1’ can be extracted from the dependency syntactic graph. The nodes and edges on the SDP denote the tokens and dependency relations on the SDP between ‘Drug0’ and ‘Drug1’, respectively

The dependency syntax information is valuable and informative for DDI extraction task. Recent studies (Liu *et al.*, 2015; Miwa and Bansal, 2016; Xu *et al.*, 2015) have shown that the dependency path or syntax tree can boost the performance of the relation extraction. Figure 1 is an illustration example of SDP. We use the Stanford parser to get the dependency syntax relations and part-of-speech (POS) of each word in the candidate sentence. For example, ‘administered/VBN’ denotes that the POS of the word ‘administered’ is ‘VBN’, whereas ‘nsubjpass’ denotes the dependency relation between ‘administered’ and ‘regimen’ is ‘nsubjpass’ type. ‘Drug0’ and ‘Drug1’ denote two targeted drug entities, respectively. The tokens and dependency relations on the shortest path between two targeted entities are shown in bold. Based on the dependency relations, the SDP between the two targeted entities is obtained, which only keep the vital words on the syntax path between two entities while filtering out the less important adjunct word (e.g. ‘to’ and ‘with’). In Figure 1, the sentence consists of multiple clauses, but we can determine the relation between ‘Drug0’ and ‘Drug1’ accurately, based on the information of SDP. Therefore, DDI extraction will benefit from the syntactic and semantic information of SDP, especially for the long and complicated sentences.

### 2.2 Hierarchical RNNs model

**Fig. 2.**
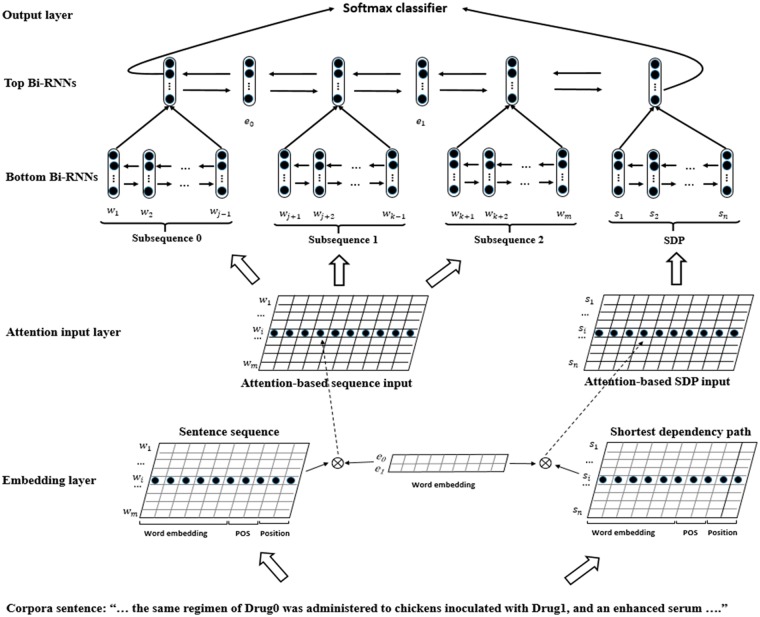
The overview of our hierarchical RNNs model on sequence and SDP

Most DDI extraction studies only use the sentence sequence as the input of neural networks (Liu *et al.*, 2016; Sahu and Anand, 2017). Although the neural networks are able to learn the feature from the sentence sequence directly, it is still hard to obtain enough lexical, syntactic and semantic cues necessary to detect and classify the DDI accurately. We propose an input level attention-based hierarchical RNNs model to integrate the SDP with sentence sequence. The schematic overview of our model is shown in the Figure 2. The input layer consists of the sentence sequence and the SDP, which are encoded by using word vector embedding, POS embedding and position embedding. An input attention mechanism is employed to capture the relevance of word with respect of the targeted drug entities. Then, the sentence sequence is divided into five parts including three context subsequences and two targeted drug entities based on the position of the targeted drug entities. Hierarchical bidirectional RNNs model is used to learn the feature representation from subsequences, SDP and targeted drug entities. Finally, the feature representation learned from the sentence sequence and SDP will be fed to Softmax function in the output layer for the DDI detection and classification. The remainder of this section will introduce the further details about our model.

#### 2.2.1 Embedding input representation

The input of our model are sentence sequence and SDP. Given a sentence *S*, $\left\{w_{1},w_{2},\ldots ,w_{m}\right\}$ and $\left\{s_{1},s_{2},\ldots ,s_{n}\right\}$ denote the sentence sequence and SDP. Each word $w_{i}$ on the sentence sequence and $s_{j}$ on the SDP are represented by word vector embedding, POS embedding and position embedding, respectively.

Bengio *et al.* (2003) proposed word embedding method using neural networks, which was one of the important results achieved by neural networks in the NPL domain. Word embedding maps words to low-dimensional real space and captures the semantic information underlying the words. In the past decade, various word embedding methods (Mikolov *et al.*, 2013; Pennington *et al.*, 2014) have been proposed for learning language models. Recently, word embedding has successfully applied to NLP tasks and achieved the state-of-the-art performance, such as information retrieval (Palangi *et al.*, 2016), relation extraction (Zeng *et al.*, 2014), machine translation (Zou *et al.*, 2013) and so on. Besides word embeddings, we also exploit POS embedding and position embedding to extend the input representation ability. The POS embedding reflects the POS feature of the words, which is valuable for DDI extraction (Zhao *et al.*, 2016). The position embedding captures the position feature and distinguishes the relative distances between each word and the targeted drug entities (Zeng *et al.*, 2014).

In our experiments, we use the abstracts containing the key word ‘drug’ from PubMed as the training corpus and employ word2vec (Mikolov *et al.*, 2013) to train the word embedding and POS embedding. For position embedding, we randomly initialize the position vector following standard normal distribution, as reported elsewhere (Wang *et al.*, 2016). Let $W_{word}$,$\;W_{POS}$ and $W_{dis}$ denote the word embedding matrix, POS embedding matrix and position matrix, respectively. Given a word $w_{i}$ on the sentence sequence, we can obtain the word embedding vector $w_{i}^{word}$, the POS embedding vector $w_{i}^{POS}$, and two position vectors $w_{i}^{dis0}$ and$\;w_{i}^{dis1}$, respectively, based on $W_{word}$,$\;W_{POS}$ and $W_{dis}$. Here, $w_{i}^{dis0}$ and$\;w_{i}^{dis1}$ are the two position vector of *i* with regard to two targeted drug entities $e_{0}$ and $e_{1}$. Thus, the overall word embedding representation for word $w_{i}$ is $z_{wi}=[{\left(w_{i}^{word}\right)}^{T},{\left(w_{i}^{POS}\right)}^{T},{\left(w_{i}^{dis0}\right)}^{T},{\left(w_{i}^{dis1}\right)}^{T}]$. Similarly, for a given word $s_{j}$ on the SDP, the overall word embedding representation is $z_{sj}=[{\left(s_{j}^{word}\right)}^{T},{\left(s_{j}^{POS}\right)}^{T},{\left(s_{j}^{dis0}\right)}^{T},{\left(s_{j}^{dis1}\right)}^{T}]$.

For DDI extraction, the drug entity generally contains one or a few words. Let e denote a drug entity in the sentence *S*. The drug entity *e* contains *l* words $\left\{w_{i},\ldots ,w_{i+l-1}\right\}$. In our experiments, we consider the drug entity *e* as a whole. The word embedding vector of drug entity *e* is mean value of *l* words embedding vectors $e^{word}=(\sum_{i}^{i+l-1}w_{i}^{word})/l$. The POS of drug entities are set as noun. Thus, the overall entity embedding representation of *e* is $z_{e}=[{\left(e^{word}\right)}^{T},{\left(e^{POS}\right)}^{T},{\left(e^{dis0}\right)}^{T},{\left(e^{dis1}\right)}^{T}]$.

#### 2.2.2 Entity attention mechanism

In general, the importance of different words in a sentence is generally different for the DDI extraction task. Consider that in a long sentence consisting of multiple clauses, the key words for determination of DDI is likely to be only a few nouns and verbs. However, each input word shares the same weight in the input layer of neural networks, which cannot distinguish the importance of different words. It is more reasonable to assign the weight for each word according to its contribution or importance to DDI extraction. Therefore, we use the entity attention mechanism to automatically learn the weight for each input word, as proposed by (Wang *et al.*, 2016).

Intuitively, the relevance of the word with respect of two drug entities can reflect the importance of the word for DDI extraction. The word embedding vector can effectively represent the hidden semantic information of the word (Mikolov *et al.*, 2013). We can calculate the semantic relevant between two words using the dot product of their word embedding vectors. For a word $w_{i}$ on the sentence sequence, the relative relevance degree with regard as drug entity $e_{k}$ (k∈{0, 1}) is defined as follow:

(1) $$\theta_{wi}^{k}=\frac{exp(dot(w_{i}^{word},\;{\;e}_{k}^{word}))}{\sum_{l=1}^{m}exp((dot(w_{l}^{word},\;{\;e}_{k}^{word}))}$$

Based on the two relevance factors $\theta_{wi}^{0}$ and $\theta_{wi}^{1}$, the joint weight for word $w_{i}$ is calculated as a simple average. The attention vector representation of the word $w_{i}$ is defined as follow:

(2) $$z_{wi}^{att}=\frac{\theta_{wi}^{0}+\theta_{wi}^{1}}{2}{\cdot z}_{wi}\;$$

The words on both sentence sequence and SDP are mapped to the attention vector representation using Equations (1) and (2). As shown in Figure 2, we use ‘Attention-based sequence input’ and ‘Attention-based SDP input’ to represent the sentence sequence matrix [$z_{w1}^{att},z_{w2}^{att},\ldots ,z_{wm}^{att}$] and the SDP matrix [$z_{s1}^{att},z_{s2}^{att},\ldots ,z_{sn}^{att}$], respectively.

#### 2.2.3 Hierarchical bidirectional LSTMs

Given a sentence sequence with two targeted entities, most studies consider the sentence sequence and the two targeted entities as a whole part. Some recent studies (Mingguang Xiao, 2016; Vu *et al.*, 2016) explored to divide the sentence sequence into multiple parts for relation extraction task and achieved excellent performance. Some sentences in biomedical texts are very long and more complicated, which are hard to deal with for neural networks, even though for RNNs model. For instance, the longest sentence in the DDI extraction 2013 corpora contains >150 words. Inspired by the work of (Mingguang Xiao, 2016), we divide the sentence sequence into three subsequence according to the position of two targeted entities. Figure 2 shows how the sentence sequence is divided into five part including ‘subsequence 0’, ‘e0’, ‘subsequence 1’, ‘e1’ and ‘subsequence 2’. We integrate the SDP with the sentence sequence to make use of the shortest dependency information between two entities.

RNNs are powerful models for NLP tasks, and are particularly suitable for encoding sequential text data. However, the traditional RNNs suffer from the vanishing gradient problem during the model training. Since RNNs models use the values of the previous hidden states and gradients to update of the hidden states repeatedly, the operations of multiplication and differentiation generally make the gradients tend to vanish over a long time. To address this problem, long short-term memory networks (LSTMs) (Hochreiter and Schmidhuber, 1997) and gated recurrent units (GRUs) (Cho *et al.*, 2014) have been proposed based on RNNs.

The basic LSTMs model exploits the memory cell and gating mechanism to make each recurrent unit to adaptively capture dependencies over different time scales and learn long-term dependencies. The LSTMs model is introduced in the Supplementary Material. Since LSTMs model is a sequential model, a LSTMs unit will generate a hidden state $h_{j}$ and keep current memory cell $c_{j}$ at the time step *j*, which operates on the current word $x_{j}$, the previous hidden state $h_{j-1}$ and the previous memory cell $c_{j-1}$. The bidirectional LSTMs (Bi-LSTMs) model consists of the forward LSTMs and backward LSTMs. This makes the Bi-LSTMs model has the ability to read the sequential input {$x_{1},x_{2},\ldots ,x_{k}$} not only from $x_{1}$ to $x_{k}$ but also from $x_{k}$ to $x_{1}$, and learn more comprehensive feature than one way LSTMs model. The output ${\;h}_{k}^{f}$ and ${\;h}_{k}^{b}$ of the forward LSTMs and backward LSTMs will be concatenated into $h_{k}$=${\;h}_{k}^{f}{\;||h}_{k}^{b}$ which is the output vector of Bi-LSTMs.

In this study, a hierarchical Bi-LSTMs model is employed to automatically learn the feature representation for the DDI extraction task. As shown in Figure 2, the hierarchical Bi-LSTMs model contains bottom Bi-LSTMs and top Bi-LSTMs. The bottom Bi-LSTMs are used to independently learn feature representations from three subsequences and SDP, respectively. The input of the bottom LSTMs are the attention-based vector representations of three subsequences and SDP. The output of the bottom LSTMs are the feature representations of the three subsequences and SDP. The top Bi-LSTMs are applied to integrate the semantics and syntax information of three context subsequence, two targeted entities and SDP, which learn the feature representation of the whole sentence and SDP.

#### 2.2.4 Classification and training

In the output layer, the feature representation *s* generated by the hierarchical Bi-LSTMs model will be fed to a fully connected neural layer in which the number of output nodes equals to the number of DDI types. Softmax function is employed as the activation function of the output layer to implement the detection and classification of DDI. The probability value of the candidate DDI belonging to the *i* type category is calculated as follow:

(3) $$p(i|s)=\;softmax(W_{o}\cdot s+b_{o})$$

where $W_{o}$ and $b_{o}$ are the weight parameters, and *s* is the feature representation of the candidate DDI. Our model uses cross-entropy cost function as the training objective function. Resilient mean square propagation (RMSProp) is used to optimize the parameters of our model with respect of the objective function.

## 3 Results and discussions

### 3.1 Datasets and experimental settings

DDI extraction 2013 corpus (Herrero-Zazo *et al.*, 2013; Segura-Bedmar *et al.*, 2014) is a manually annotated DDI corpus based on the DrugBank and MedLine abstracts. The DDI extraction 2013 corpus is the major corpus to evaluate and compare the performance of DDI extraction methods. The original DDI 2013 corpus contains 714 train files and 191 test files. There are 90 train files which have no relevance to DDI in the 714 train files. In our experiments, we use 624 train files and 191 test files to evaluate the performance of our method.

The DDI 2013 corpus contains four DDI types: *Advice*, *Effect*, *Mechanism* and *Int*. *Advice* is used to annotate the semantic relation describing an advice or recommendation regarding a drug interaction. *Effect* is used to annotated the semantic relation describing an effect or pharmacodynamics mechanism. *Mechanism* is used to annotated the semantic relation about pharmacokinetic mechanism. *Int* is used to annotated the semantic relation without any further information is mentioned. The DDI extraction model detects the DDI as well as classifies the DDI with the correct DDI type. The detailed statistics of DDI extraction 2013 corpus is listed in Table 1.

**Table 1.** The statistics of the DDI 2013 extraction corpus

Corpus	Advice	Effect	Mechanism	Int	Negative
Training set	826	1687	1319	188	23772
Test set	221	360	302	96	4737
Total	1047	2047	1621	284	28554

Most of the DDI extraction methods use *F*-score, *precision* and *recall* as the evaluation metrics. To keep the same metrics with the existing methods, we also use *F-*score, *precision* and *recall* to evaluate the performance of our method. The *F-*score is defined as (2·precision·recall)/(precision+recall), which can quantify the overall performance by balancing the *precision* and *recall*.

In our experiments, we use Keras library to implement our proposed model. The dimensionality of word embedding, POS embedding and position embedding is 200, 10 and 10, respectively. Due to the computation reason of neural networks model, it is not possible to search the optimal value for the hyper-parameters of our model. We manually tune the hyper-parameters using 5-fold cross-validation on the training set. The hyper-parameters after tuning used in our experiments are as follows. The hidden unit number of bottom LSTMs and top LSTMs are both 100, the learning rate of RMSProp is set as 0.001, and the mini-batch size is set as 64. Neural networks models generally contain a large number of parameters and suffer from the overfitting problem. Dropout is an effective way to alleviate the overfitting of the neural networks model (Srivastava *et al.*, 2014), which randomly drops units and their connections from the neural networks during training. In our experiments, we apply dropout on the embedding layer and output layer. The dropout rate of embedding layer and output layer are set as 0.7 and 0.5, respectively.

### 3.2 Experimental results

In this section, we first evaluate the effectiveness of different RNNs. The simple RNNs model is a traditional RNNs architechture, which do not contain logical gate and memory cell. The GRUs model (Cho *et al.*, 2014) exploits the gating mechanism to make each recurrent unit to adaptively capture dependencies over different time scales, but does not contain a memory cell. The LSTMs model (Hochreiter and Schmidhuber, 1997) employs a gating mechanism as well as the memory cell to learn long-term dependencies. The comparison performance of simple RNNs, GRUs and LSTMs for our method are listed in Table 2.

**Table 2.** The evaluation of different RNNs model on performance

RNNs model	Precision	Recall	*F*-score	Δ
Simple RNNs	0.657	0.576	0.614	
GRUs	0.733	0.715	0.724	+0.11
LSTMs	0.741	0.718	0.729	+0.05

*Note*: ‘Δ’ denotes the corresponding improvement of *F*-score.

Table 2 shows the results of using different RNNs. Simple RNNs model can only achieve a *F*-score of 0.614 while GRUs and LSTMs achieve a higher *F*-score of 0.724 and 0.729, respectively. The experimental results suggest that the logical gate and memory cell can help LSTMs model to capture more syntactic feature or information over long-term scales, which are beneficial for determination of DDI relation in the sentence.

Then, we evaluate the effectiveness of embedding feature of our method. The experimental results are shown in Table 3. Our method achieves an *F*-score of 0.703 when only using word embedding in the embedding layer. When POS embedding and position embedding are integrated with word embedding, the performance is further improved. These results show that the word embedding contributes to the success of the DDI extraction task, as only using word embedding can also achieve a high *F*-score. Moreover, the POS embedding and position embedding are valuable supplemental features for DDI extraction task.

**Table 3.** The effect of the embedding feature on performance

Embedding feature	Precision	Recall	*F*-score	Δ
Word	0.688	0.717	0.703	
Word+POS	0.717	0.713	0.715	+0.12
Word+POS+Position	0.741	0.718	0.729	+0.14

*Note*: ‘Word’, ‘POS’ and ‘Position’ denote word embedding, POS embedding and position embedding, respectively. ‘Δ’ denotes the corresponding improvement of *F*-score.

Next, we compare with the baseline method to evaluate the effectiveness of our model. (Sahu and Anand, 2017) proposed bidirectional LSTMs model (B-LSTMs) and joint LSTMs model (Joint-LSTMs) for DDI extraction task. B-LSTMs model use bidirectional LSTMs and max pooling on the sentence sequence. Joint-LSTMs integrate two B-LSTMs models and attention pooling on the sentence sequence. The comparison performance with B-LSTMs and Joint-LSTMs is shown in Table 4. When Bi-LSTMs model is employed on sentence sequence and SDP, we achieve *F*-score of 0.696 and 0.526, respectively. The sentence sequence contains all the words, whereas SDP only keep the vital words of the sentence. Hence, the sentence sequence contains richer lexical and syntactic information than SDP. This is the mainly reason why Bi-LSTMs model achieves higher performance on sentence sequence than SDP. When the hierarchical Bi-LSTMs method is employed on sentence sequence, the *F*-score improves from 0.696 to 0.707. This suggests that the hierarchical Bi-LSTMs can capture more valuable features by dividing the sentence into three subsequences, and improve the performance effectively. When the embedding attention mechanism is added, the *F*-score improves to 0.717. This indicates that the embedding attention can identify and enhance the weight of the key words in the sentence, which further improves the performance of RNNs model for DDI extraction. Furthermore, our model achieves a *F*-score of 0.729, when integrating the SDP with sentence sequence. The improvement of performance benefits from the vital syntactic and semantic information of the SDP, which is valuable for the relation of the two candidate drug entities. Compared with (Sahu and Anand, 2017), our method achieves superior *F*-score of 0.729 based on integrating SDP and embedding attention. Beside of *F*-score, *precision* and *recall*, we also provide the confusion matrix of our results in the Supplementary Material.

**Table 4.** The effect of strategy on performance

Model	Precision	Recall	*F*-score
B-LSTMs (Sahu and Anand, 2017)	0.76	0.656	0.704
Joint-LSTMs (Sahu and Anand, 2017)	0.734	0.697	0.715
SDP Bi-LSTMs	0.592	0.474	0.526
Sequence Bi-LSTMs	0.702	0.691	0.696
Hierarchy Bi-LSTMs	0.725	0.689	0.707
Hierarchy Bi-LSTMs +Att.	0.73	0.703	0.717
Hierarchy Bi-LSTMs +Att.+SDP	0.741	0.718	0.729

*Note*: ‘Att.’ denotes using embedding attention mechanism.

### 3.3 Performance comparison with state-of-the-art methods

In this section, we compare our method with other state-of-the-art methods on DDI 2013 corpus. In Table 5, we compare the overall performance and each DDI type. Neural networks-based methods generally achieve better performance than feature-based methods and kernel-based methods. For example, (Quan *et al.*, 2016) employed multichannel CNNs model and achieved the highest precision of 0.76 and a high *F*-score of 0.702, respectively. (Sahu and Anand, 2017) used LSTMs model with attention pooling and achieved an *F*-score of 0.715. We also notice that (Raihani and Laachfoubi, 2016) used rich feature-based method and achieved a high *F*-score of 0.711, which benefited from many rules and handcraft features. Compared with feature-based method, Neural networks-based methods not only learn the feature representation from the sentence automatically but also achieve state-of-the-art performance. This indicates the effectiveness and potential of the neural networks-based methods for DDI extraction. Among the neural networks-based methods, CNNs and RNNs are the two models commonly used for DDI extraction task. Yin *et al.* (2017) compared the performance between CNNs and RNNs on NLP tasks systematically. The comparison results have shown that the performance of CNNs and RNNs are very close for the relation classification task on SemEval 2010 corpus (Hendrickx *et al.*, 2009). However, some studies (Sahu and Anand, 2017; Yi *et al.*, 2017) also suggested that RNNs models achieved higher performance than CNNs models on DDI 2013 corpus. The mainly reason is that the DDI 2013 corpus is based on DrugBank and MedLine, and contains many long and complicated sentences. Compared with CNNs, RNNs model can effectively learn the long-term dependence of the sentence, which is vital to capture the lexical and syntactic feature in the long and complicated sentence for the relation extraction task. Our method exploits hierarchical Bi-LSTMs to integrate the sentence and SDP, and the embedding attention mechanism to identify and enhance key words of the candidate sentences. The strategy can further improve the ability of RNNs model to deal with the long and complicated sentences. Our method achieves the highest *F*-score of 0.729 and recall of 0.718, respectively. (Yi *et al.*, 2017) proposed a GRUs-based method to extract DDI and employed multiple layer attention to boost the performance, which achieved precision, recall and *F*-score of 0.737, 0.708 and 0.722, respectively. The high *F*-score of 0.722 (Yi *et al.*, 2017) is only inferior to our method, and outperforms other methods, which benefits from the word level attention and sentence level attention. Both our results and (Yi *et al.*, 2017) indicate that the attention mechanism can improve the performance for DDI extraction effectively.

**Table 5.** Performance comparison with other state-of-the-art methods on DDI extraction 2013 corpus

	Methods	*F*-score on each DDI type				Overall performance		
		Advice	Effect	Mechanism	Int	Precision	Recall	*F*-score
Feature-based methods	UTurku (Björne *et al.*, 2013)	0.63	0.6	0.582	0.507	0.732	0.499	0.594
	(Kim *et al.*, 2015)	0.725	0.662	0.693	0.483	—	—	0.67
	(Raihani and Laachfoubi, 2016)	0.774	0.696	0.736	0.524	0.737	0.687	0.711
Kernel-based methods	FBK-irst (Chowdhury and Lavelli, 2013)	0.692	0.628	0.679	**0.547**	0.646	0.656	0.651
	WBI (Thomas *et al.*, 2013)	0.632	0.61	0.618	0.51	0.642	0.579	0.609
	Zheng *et al.* (2016)	0.714	0.713	0.669	0.516	—	—	0.684
Neural networks-based methods	SCNN (2016)	—	—	—	—	0.725	0.651	0.686
	Quan *et al.* (2016)	0.782	0.682	0.722	0.51	**0.76**	0.653	0.702
	Liu *et al.* (2016)	0.777	0.693	0.702	0.464	0.757	0.647	0.698
	Joint-LSTMs (Sahu and Anand, 2017)	0.794	0.676	**0.763**	0.431	0.734	0.697	0.715
	Yi *et al.* (2017)	—	—	—	—	0.737	0.708	0.722
	Our method	**0.803**	**0.718**	0.74	0.543	0.741	**0.718**	**0.729**

*Note*: The highest value is shown in bold. The ‘—’ denotes the value is not provided in the paper.

Then, we compared the performance on each DDI type. Our method achieves the highest *F*-score on *advice* and *effect* types, whereas Joint-LSTMs and FBK-irst achieve the highest *F*-score on *mechanism* and *int* type, respectively. As a whole, the performance on different DDI type vary significantly. On *advice* type, all methods achieve relatively high performance. On the contrary, all the *F*-score on *int* type are no >0.6. This suggests that it is the most difficult to accurately extract *int* type DDI on the DDI extract 2013 corpus. From Table 1, we can see that the training set for *int* type only contain 188 instances which is far less than other DDI types. The sufficient training data is crucial for the performance of both statistical machine learning-based models and neural networks-based models. The insufficient training data for *int* type will lead the under fitting of the models. This is probably the major reason for the worse performance on *int* type.

In addition, we perform error analysis for the false negatives in the Supplementary Material.

Overall, the performance comparison shows that our method is competitive or superior in performance, compared with other state-of-the-art methods used for DDI extraction.

## 4 Conclusions

The SDP contains valuable syntactic and semantic information for the DDI extraction task. However, most neural networks-based methods only use the sentence sequence as the input of the models, which limits the performance of DDI extraction task. In this paper, we present a hierarchical RNNs model to integrate the SDP of candidate sentence with the sentence sequence for DDI extraction task. We divide the sentence sequence into three parts according to the position of two entities, and apply a hierarchical RNNs model to integrate sentence sequence and SDP for DDI extraction. Furthermore, we introduce an embedding attention mechanism to identify and enhance the key words which exist the close semantic relation with regard of two entities. Experimental results show that hierarchical RNNs model can effectively integrate SDP with sentence sequence, and improve the performance for DDI extraction. It is encouraging to see that our method achieves the highest *F*-score of 0.729 on the DDI 2013 corpus, which outperforms other state-of-the-art methods.

Although our method has achieved the best performance on DDI 2013 corpus, there is still some room to improve. In particular, our method does not perform well on the *int* type, likely because of insufficient training data. This indicates that our method depends on the high quality training data. As future work, we aim to develop new human-computation approaches to increase the amount and quality of training data. In addition, we also plan to employ semi-supervised method for biomedical relation extraction.

## Supplementary Material

Supplementary Data
