# Supplementary material for: Drug–drug interaction extraction via hierarchical RNNs on sequence and shortest dependency paths
Source: Bioinformatics. 2017 Oct 25;34(5):828–35. doi: 10.1093/bioinformatics/btx659 (PMC6030919; doi:10.1093/bioinformatics/btx659)
Supplement: Supplementary Data [file btx659_supplementary_materials.docx]

Supplementary Materials

**Supplementary Material:** Illustration of DDI extraction 2013 corpus

DDI 2013 corpus is annotated and built by ([Herrero-Zazo, et al., 2013](#_ENREF_1); [Segura-Bedmar, et al., 2014](#_ENREF_5)), which is distributed in XML documents following the format ([Pyysalo, et al., 2008](#_ENREF_3)). Fig. S1 is a fragment from the DDI 2013 corpus. The “entity” denotes drug entity and “pair” denotes the DDI in Fig. S1. It can be seen that the sentence “DDI-DrugBank.d31.s0” contains two drug entities, “MAO inhibitors” and “hydralazine”. DDI 2013 corpus proposed four entity types to annotate pharmacological substances including drug (generic drug names), brand (branded drug names), group (drug group names) and drug_n (active substances not approved for human use). The relation between “MAO inhibitors” and “hydralazine” is annotated as “advise” type. The DDI 2013 corpus contains four DDI types: *Advice*, *Effect*, *Mechanism* and *Int*. *Advice* is used to annotate the semantic relation describing an advice or recommendation regarding a drug interaction. *Effect* is used to annotated the semantic relation describing an effect or pharmacodynamics mechanism. *Mechanism* is used to annotated the semantic relation about pharmacokinetic mechanism. *Int* is used to annotated the semantic relation without any further information is mentioned.


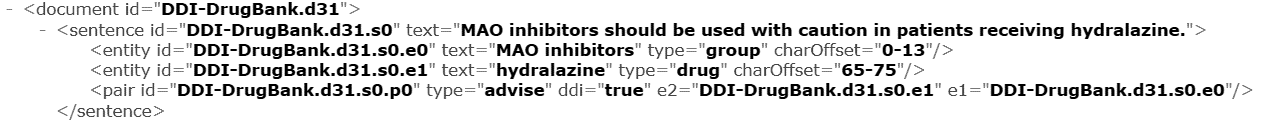


**Fig. S1. An example of DDI 2013 corpus.**

**Supplementary Material:** confusion matrix of our results

Table S1 shows the confusion matrix of the results of our model. It can be seen that *Advice*, *Effect*, *Mechanism* type DDIs are often misclassified to *Negative* type. From Table 1, we can see that the positive instances and the negative instances are significantly imbalance. There are 4020 positive DDIs and 23772 negative DDIs in the training data set. The imbalance of the training data will increase the risk of misclassifying to Negative type. We also notice that *Int* type DDIs are often misclassified to *Effect* type. This experimental results is similar to ([Sahu and Anand, 2017](#_ENREF_4); [Yi, et al., 2017](#_ENREF_6)). It indicates that it is the most difficult for our model to accurately distinguish *int* type DDIs from *Effect* type.

**Table S1.**The confusion matrix of our model results

| Type | Advice | Effect | Mechanism | Int | Negative |
| --- | --- | --- | --- | --- | --- |
| Advice | 167 | 6 | 3 | 2 | 43 |
| Effect | 2 | 284 | 7 | 0 | 67 |
| Mechanism | 8 | 6 | 211 | 0 | 77 |
| Int | 0 | 40 | 2 | 41 | 13 |
| Negative | 18 | 95 | 45 | 12 | 4567 |

**Supplementary Material:** RNNs and LSTMs model

Recurrent neural networks (RNNs) are deep neural networks which contain a series of temporal units. RNNs model can use their internal memory to capture long sequence of inputs. This makes RNNs model are very suitable for various of natural language processing (NLP) tasks. Fig. S2 shows the typical RNNs models architecture. The $x_{0},x_{1},\ldots,x_{j}$ and $h_{0},h_{1},\ldots,h_{j}$ are the inputs and outputs of RNN models. The architecture of RNNs allows the input information to be passed from one unit to the next unit.


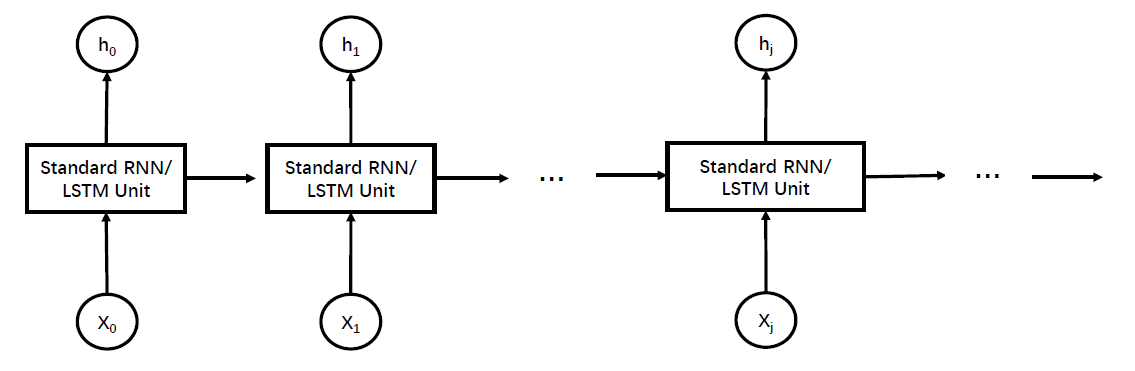


**Fig. S2. RNNs models architecture.**

The standard RNNs suffer from the vanishing gradient problem during the model training. Since RNNs model uses the values of the previous hidden states and gradients to update of the hidden states repeatedly, the operations of multiplication and differentiation generally make the gradients tend to vanish over a long time. This makes standard RNNs cannot completely capture the long-term dependencies in practice. To address this problem, ([Hochreiter and Schmidhuber, 1997](#_ENREF_2)) proposed long short-term memory networks (LSTMs) that employs gating mechanism to alleviate the vanishing gradient problem.


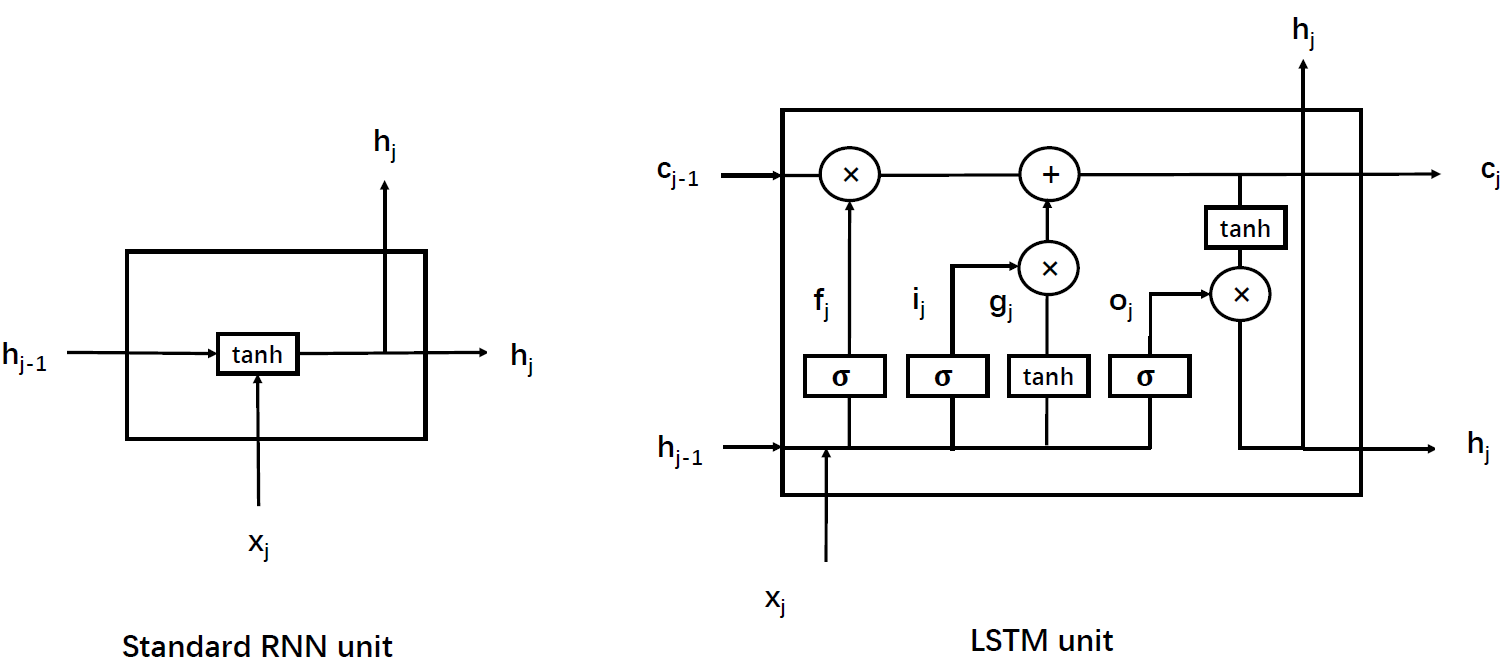


**Fig. S3. Standard RNNs and LSTMs unit architectures.**

Fig. S3 shows the standard RNNs and LSTMs unit architectures. For standard RNNs model, the repeating unit is a simple structure, which only contain an activation function such as tanh. The LSTMs model exploits the memory cell and gating mechanism to make each recurrent unit to adaptively capture dependencies over different time scales and learn long-term dependencies. A LSTMs unit will generate a hidden state $h_{j}$ and keep current memory cell $c_{j}$ at the time step j, which operates on the current word $x_{j}$, the previous hidden state $h_{j-1}$ and the previous memory cell $c_{j-1}$. The LSTMs address the vanishing gradient problem by employing gate mechanism including input gate, forget gate and output gate. The input gate determines whether the input will be stored in the memory cell. The forget gate determines whether current contents of memory will be forgotten. The output gate determines whether current memory contents will be output. At the time step *j*, the input gate $i_{j}$, the forget gate $f_{j}$, the output gate $o_{j}$ and the extracted feature vector $g_{j}$ are defined as follows, respectively.

$i_{j}=sigmoid(W_{i}x_{j}+U_{i}h_{j-1}+b_{j})$(1)

$f_{j}=sigmoid(W_{f}x_{j}+U_{f}h_{j-1}+b_{f})$(2)

$o_{j}=sigmoid(W_{o}x_{j}+U_{o}h_{j-1}+b_{o})$(3)

$g_{j}=tanh(W_{g}x_{j}+U_{g}h_{j-1}+b_{g})$(4)

Based on above definitions, the memory cell $c_{j}$ and hidden state $h_{j}$ at the time step j can be calculated as follows:

$c_{j}=f_{j}{\bigotimes c}_{j-1}+i_{j}\bigotimes g_{j}$(5)

$h_{i}=o_{j}\bigotimes{tanh(c}_{j-1})$(6)

In the equation (1)~(6), $W_{*}$ , $U_{*}$ are the weight matrices, $b_{*}$ are bias vectors, and $\bigotimes$ denotes element wise multiplication.

**Supplementary Material:** error analysis for false negatives

In this section, we manually analyzed what sentences lead to false negatives, since those are more critical than false positives. Fig. S4 shows some examples of false negatives. The two drug entities are in bold. Most false negatives are caused by coordinate structures and appositions. In the future plan, further studies should be performed on how to identify the DDI on coordinate structures and appositions accurately. Another frequent cause of false negatives is that the two drug entities are in different clauses. For example, in FN4, the two drug entities “cisapride” and “macrolide antibacterials” are in different clauses, which are over long distance with each other. In this case, it is hard for the model to distinguish the relation between “cisapride” and “macrolide antibacterials”.


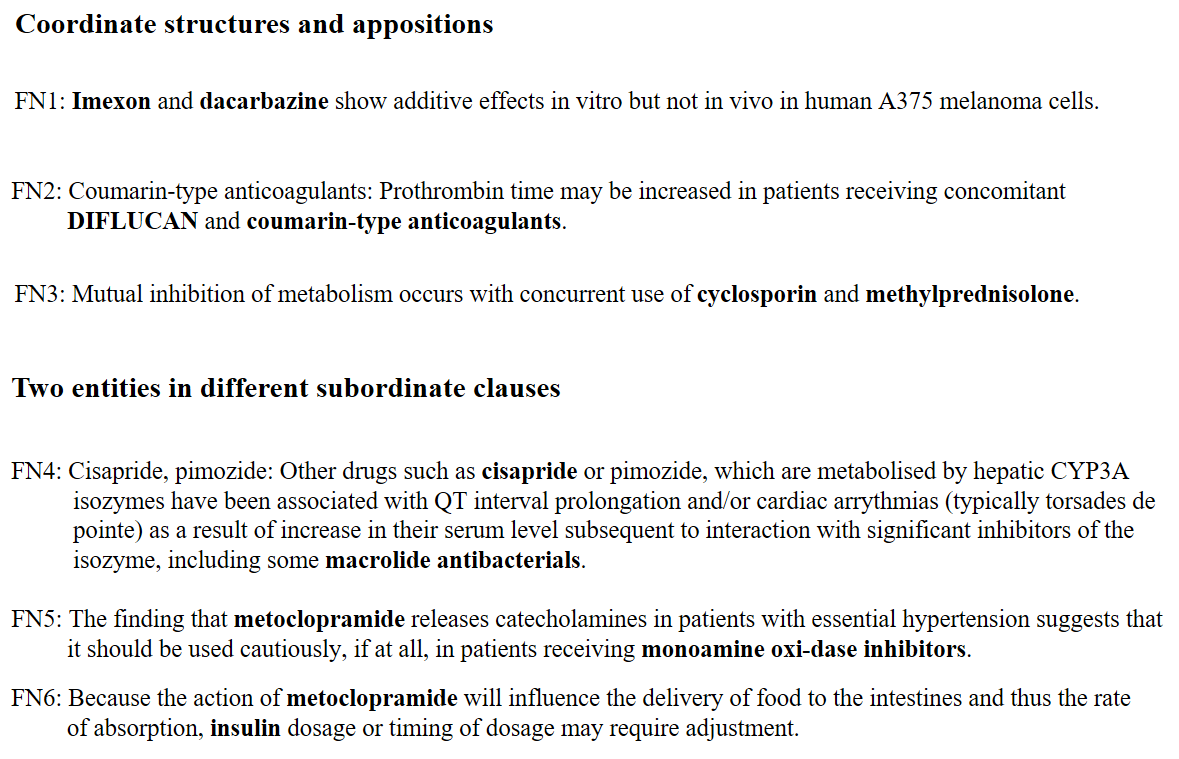


**Fig. S4. Examples of false negatives.**

**Reference**

Herrero-Zazo, M.*, et al.* (2013) The DDI corpus: An annotated corpus with pharmacological substances and drug–drug interactions, *Journal of biomedical informatics*, **46**, 914-920.

Hochreiter, S. and Schmidhuber, J. (1997) Long short-term memory, *Neural computation*, **9**, 1735-1780.

Pyysalo, S.*, et al.* (2008) Comparative analysis of five protein-protein interaction corpora, *BMC bioinformatics*, **9**, S6.

Sahu, S.K. and Anand, A. (2017) Drug-Drug Interaction Extraction from Biomedical Text Using Long Short Term Memory Network, *arXiv preprint arXiv:1701.08303*.

Segura-Bedmar, I., Martínez, P. and Herrero-Zazo, M. (2014) Lessons learnt from the DDIExtraction-2013 shared task, *Journal of biomedical informatics*, **51**, 152-164.

Yi, Z.*, et al.* (2017) Drug-drug Interaction Extraction via Recurrent Neural Network with Multiple Attention Layers, *arXiv preprint arXiv:1705.03261*.
